# A Protocol for the Production of KLRG1 Tetramer

**DOI:** 10.3791/1701

**Published:** 2010-01-12

**Authors:** Stephanie C. Terrizzi, Cindy Banh, Laurent Brossay

**Affiliations:** Department of Molecular Microbiology and Immunology, Brown University

## Abstract

Killer cell lectin-like receptor G1 (KLRG1) is a type II transmembrane glycoprotein inhibitory receptor belonging to the C type lectin-like superfamily. KLRG1 exists both as a monomer and as a disulfide-linked homodimer. This well-conserved receptor is found on the most mature and recently activated NK cells as well as on a subset of effector/memory T cells.

Using KLRG1 tetramer as well as other methods, E-, N-, and R-cadherins  were identified as KLRG1 ligands. These Ca^2+^-dependent cell-cell adhesion molecules comprises of an extracellular domain containing five cadherin repeats responsible for cell-cell interactions, a transmembrane domain and a cytoplasmic domain that is linked to the actin cytoskeleton.

Generation of the KLRG1 tetramer was essential to the identification of the KLRG1 ligands. KLRG1 tetramer is also a unique tool to elucidate the roles cadherin and KLRG1 play in regulating the immune response and tissue integrity.

**Figure:**
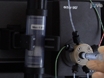


## Protocol

### Preparation of Inclusion Bodies

Inoculate 4 x 500 ml flasks of TB each containing 50 μg/ml of carbenicillin and chloramphenicol with an overnight culture of bacteria expressing the KLRG1 plasmid construct.  When the OD reaches 0.6 Å at 600 nm, induce with the addition of 0.4 M IPTG and incubate the culture for an additional 4 hours shaking at 37°C.
Resuspend the harvested cells in resuspension buffer pH=8.0 (50 mM Tris-HCl, 25% (W/V) sucrose, 1 mM EDTA, 10 mM DTT).  Note: pellets can be frozen at this stage.
Pool no more than 60 ml of bacterial resuspensions in a polypropylene beaker. If necessary adjust to 60 ml with TE buffer pH 8.0 and stir mixture at half speed.
To stirring mixture add drop wise: lysozyme (final=1 mg/ml), MgCl2 (final=5 mM), 2 mg DNAse I in 50% glycerol contained 75 mM NaCl, Triton-X100 (final=1%), DTT (final=10 mM).
Sonicate the solution on ice for 1.5 min and centrifuge the lysates at 5,000 RPM for 10 min at 4°C.
  Decant the supernatant.
  Add 15 ml of wash buffer pH=8.0 (50 mM Tris-HCl, 0.5% Triton X-100, 100 mM NaCl, 1 mM EDTA, 1 mM DTT).
  On ice, sonicate solution for 1.5 min until the pellet is completely resuspended.
  Centrifuge the samples at 5,000 RPM for 10 min at 4°C.
  Repeat the wash 5 times.
Repeat 5b with wash buffer without Triton X-100, centrifuge and resuspend in 4 ml of TE Buffer.
Take the wet weight inclusion bodies slurry and store in TE buffer at a concentration of 30 to 100 mg/ml.

### Refolding of KLRG1

Make 1 L of refolding buffer pH=8.0 (0.4 M Arginine-HCl, 0.1 M Tris pH 8-8.3, 2 mM EDTA, 0.2 mM PMSF, 3 EDTA-free protease inhibitor tablets, 5 mM GSH and 0.5 mM GSSG) and chill to 4°C while stirring.
Prepare the inclusion bodies for injection into the folding mixture:  It is ideal to make 5   10 injections each with 0.25   0.5 μM concentration so that the final concentration of the protein will be 2   3 μM.
  Melt the volume of 50 mg of inclusion bodies slurry in 7 M GnHCl with 10 mM β-mercaptoethanol.
  Keep the inclusion bodies/GnHCl mixture at 37°C for 30 - 40 min and vortex every 5 min to ensure complete melting (slurry will become clear when completely melted).
  Centrifuge at max speed for 30 min in microcentrifuge at 4°C and transfer the supernatant to a 15 ml centrifuge tube. Do not to transfer any of the small blackish pellet.
Adjust volume with injection buffer (3 M GnHCl, 10 mM NaAcetate, pH 4.2,10 mM EDTA).
Inject 1 ml of diluted inclusion bodies every hour.  When injecting, stir at high speed, add 1 ml of diluted inclusion bodies drop by drop with 2 seconds between each drop.  When injection is done, stir at low speed.
Continue overnight stirring at low speed at 4°C.

### Concentration of Refolding Reactions

Following Millipore s protocol, concentrate the 1 L of pre-filtered (0.22 μm filtered) refolding reaction using an Amicon Stirred Cell and YM10 NMWL 10K ultrafiltration membrane (Millipore) to ~10 ml.
Concentrate to ≤2 ml using a Centriplus YM-10 10K MWCO (Millipore).

### Purification of KLRG1 tetramer

Setup the AKTAFPLC at 4°C  according to GE Healthcare manuals.
Purify the monomer form of KLRG1 by size exclusion chromatography using a Sephacryl S-200 16/60 high-resolution column (GE Healthcare) in 20 mM HEPES and 150 mM NaCl. (See Figure 1).
Concentrate to 1 ml using Centriplus YM-10 10K MWCO (Millipore).
Use BirA enzyme according to Avidity s protocol to biotinylate the KLRG1 monomer.
The free biotin is eliminated by size exclusion chromatography as in 2.
KLRG1 molecule is tetramerized by mixing KLRG1 monomer with 4 fold molar excess PE conjugated streptavidin (BD Biosciences).

### Representative Results


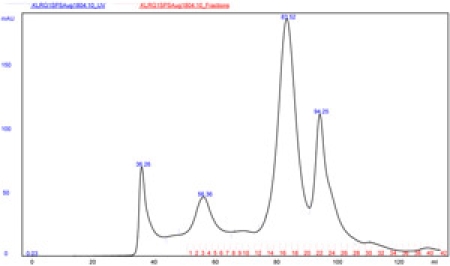
 Click here for a larger figure. **Figure 1. **Representative positive results from the AKTA FPLC size exclusion chromatography of the refolded KLRG1.  The graph shows the separation of the monomer (83.52 ml), dimer (56.36 ml), and multimer (36.26 ml) form of the refolded KLRG1.  The peak at 94.25 ml represents the buffer exchange.

## Discussion

In this protocol, it is shown how to purify, biotinylate, and tetramerize KLRG1. KLRG1 tetramer can be used to label KLRG1 ligands by flow cytometry. Although refolding conditions will have to be tested empirically for each protein, other C-type lectins could be tetramerized using a similar protocol. Using tetramerized proteins as probes, ligands to orphan C-type lectin receptors could potentially be identified.
